# Integrated Ultrasound Features, Cytologic Subclassification, and Systemic Inflammatory Markers for Predicting Malignancy in Bethesda III Thyroid Nodules

**DOI:** 10.3390/jcm15166375

**Published:** 2026-08-18

**Authors:** Emine Yildirim, Pelin Cobanoglu Akbas, Sibel Bektas, Adem Senturk, Pelin Basım, Umut Fırat Turan, Nesimi Mecit, Hanifi Onalan, Hafize Uzun, Mustafa Uygar Kalaycı

**Affiliations:** 1 Department of General Surgery, Faculty of Medicine, Atlas University, 34403 Istanbul, Türkiye; umut.turan@atlas.edu.tr (U.F.T.); nesimi.mecit@atlas.edu.tr (N.M.); hanifi.onalan@atlas.edu.tr (H.O.); mukalayci@hotmail.com (M.U.K.); 2 Department of Pathology, Gaziosmanpaşa Training and Research Hospital, University of Health Sciences, 34255 Istanbul, Türkiye; drakbaspelin@gmail.com (P.C.A.); sibel_bektas@yahoo.com (S.B.); 3 Department of Oncologic Surgery, Sakarya Training and Research Hospital, Sakarya University, 54100 Sakarya, Türkiye; dr.adem.senturk@gmail.com; 4 Department of General Surgery, Faculty of Medicine, İstanbul Medipol University, 34403 Istanbul, Türkiye; pelinakbaba@gmail.com; 5 Department of Biochemistry, Faculty of Medicine, Istanbul Atlas University, 34403 Istanbul, Türkiye; hafize.uzun@atlas.edu.tr

**Keywords:** Bethesda III, AUS/FLUS, thyroid nodule, nuclear atypia, malignancy risk, ultrasonography

## Abstract

**Background/Objectives:** Bethesda category III (atypia of undetermined significance/follicular lesion of undetermined significance; AUS/FLUS) thyroid nodules represent a heterogeneous group with variable malignancy risk, creating challenges in clinical management. This study aimed to evaluate the associations of preoperative ultrasonographic findings, inflammation-based hematological markers, and cytological atypia subtypes with final histopathological outcomes in surgically treated Bethesda III thyroid nodules. **Methods:** This retrospective study included 72 adult patients with Bethesda III thyroid nodules who underwent thyroid surgery between January 2020 and December 2025. Demographic characteristics, ultrasonographic findings, neutrophil-to-lymphocyte ratio (NLR), platelet-to-lymphocyte ratio (PLR), cytological atypia subtypes, and final histopathological diagnoses were analyzed. Univariate and multivariable logistic regression analyses were performed to identify predictors of malignancy. **Results:** Final histopathological examination revealed benign lesions in 49 patients (68.1%) and malignant lesions in 23 patients (31.9%). Age, sex, nodule size, multinodular goiter, Hashimoto thyroiditis, microcalcifications, hypoechogenicity, suspicious margins, and TI-RADS category were not significantly associated with malignancy (all *p* > 0.05). Similarly, NLR and PLR did not differ significantly between benign and malignant nodules (NLR: 1.80 vs. 1.98, *p* = 0.319; PLR: 117.9 vs. 135.0, *p* = 0.398). Among cytological atypia subtypes, nuclear atypia demonstrated the highest malignancy rate (50.0%) and was significantly associated with malignant histopathological outcomes (OR = 3.364, 95% CI: 1.182–9.570, *p* = 0.023). After adjustment for age and biopsied nodule size, nuclear atypia remained an independent predictor of malignancy (adjusted OR = 3.963, 95% CI: 1.298–12.094, *p* = 0.016). **Conclusions:** In surgically treated Bethesda III thyroid nodules, nuclear atypia was the only independent predictor of malignancy, whereas ultrasonographic characteristics, NLR, and PLR showed limited predictive value. Subclassification of Bethesda III nodules according to cytological atypia may improve preoperative risk stratification and support clinical decision-making. Because the study included only surgically treated patients, the findings should be interpreted in the context of a surgically selected cohort and may not be generalizable to the overall Bethesda III population.

## 1. Introduction

As one of the most common endocrine disorders in clinical practice, thyroid nodule detection has surged due to widespread high-resolution imaging. Although ultrasound studies identify nodules in up to 68% of adults, only a small minority are malignant. Consequently, thyroid nodule management centers on accurately identifying malignancy to safeguard patients with benign disease from unnecessary surgery, which may expose patients to surgical complications, lifelong thyroid hormone replacement, increased healthcare costs, and postoperative decision regret [1,2].

Fine-needle aspiration biopsy (FNAB) remains the gold standard for evaluating thyroid nodules. To streamline clinical management, the Bethesda System classifies FNAB findings into six distinct cytopathological categories, each with a specific malignancy risk and management protocol. Nevertheless, nodules with indeterminate cytology continue to pose a major diagnostic challenge [3].

The Bethesda III category defined as atypia or follicular lesion of undetermined significance (AUS/FLUS) remains highly controversial. It encompasses a heterogeneous group of nodules with cellular atypia insufficient for benign, suspicious, or malignant classification. Reflecting this ambiguity, reported malignancy rates vary widely from approximately 10% to 30% depending on the institution and NIFTP inclusion, with even higher rates observed in surgical cohorts [4,5]. This substantial variability complicates management and obscures the optimal clinical approach.

However, imaging-based risk stratification systems alone may not accurately predict malignancy in all patients with indeterminate thyroid cytology. Growing evidence suggests that systemic inflammation plays an important role in tumor development and progression. Accordingly, inflammation-based biomarkers derived from routine blood tests, such as the neutrophil-to-lymphocyte ratio (NLR) and platelet-to-lymphocyte ratio (PLR), have emerged as potential diagnostic and prognostic indicators in various malignancies [6]. Nevertheless, their utility in predicting malignancy among Bethesda category III thyroid nodules remains incompletely understood.

Although ultrasonographic features play an important role in malignancy risk assessment, imaging findings alone may not provide sufficient risk stratification in patients with Bethesda III thyroid nodules. In addition, inflammation-based markers such as the NLR and PLR have been proposed as potential predictors of malignancy, but their role in this subgroup remains unclear. Therefore, this study aimed to evaluate the associations of preoperative ultrasonographic findings, NLR, and PLR with final histopathological outcomes in surgically treated patients with Bethesda III thyroid nodules and to identify factors associated with malignancy that may improve preoperative risk assessment.

## 2. Materials and Methods

### 2.1. Study Design and Ethical Approval

This retrospective observational study was conducted to evaluate the association between preoperative ultrasonographic findings and final histopathological outcomes in patients with Bethesda category III thyroid nodules who underwent thyroid surgery.

The study protocol was approved by the Non-Interventional Clinical Research Ethics Committee of Istanbul Medipol University (Decision No: E-10840098-772.02-217; 5 January 2023). The study was performed in accordance with the ethical principles of the Declaration of Helsinki. Due to the retrospective design of the study and the use of anonymized patient data, the requirement for informed consent was waived.

### 2.2. Study Population

Patients who underwent thyroid surgery between January 2020 and December 2025 following a cytological diagnosis of Bethesda category III (atypia of undetermined significance/follicular lesion of undetermined significance; AUS/FLUS) were retrospectively identified from the institutional database. Patients were eligible if they were ≥18 years old, had a preoperative fine-needle aspiration biopsy classified as Bethesda category III (AUS/FLUS) according to The Bethesda System for Reporting Thyroid Cytopathology, underwent thyroid surgery, and had complete preoperative ultrasonographic and postoperative histopathological data available for review [4].

Patients were excluded if they were younger than 18 years, had a cytological diagnosis other than Bethesda category III, had a history of previous thyroid surgery or recurrent thyroid disease, lacked complete ultrasonographic records, had missing clinicopathological data, or did not have a definitive postoperative histopathological diagnosis. Only patients who met all inclusion criteria and none of the exclusion criteria were included in the final analysis.

### 2.3. Ultrasonographic Evaluation

Preoperative thyroid ultrasonography examinations were reviewed using archived radiology reports. The evaluated ultrasonographic characteristics included nodule size, echogenicity, margin characteristics, calcification status, shape, and TI-RADS category when available. TI-RADS classification was available for 43 of the 72 patients (59.7%). In the remaining 29 patients (40.3%), TI-RADS classification was not documented in the original radiology reports because standardized TI-RADS reporting was not routinely implemented during the study period. For statistical analysis, TI-RADS categories were grouped as TI-RADS 3 and TI-RADS 4. The TI-RADS 4 category included both TI-RADS 4A and TI-RADS 4B nodules because of the limited number of cases in each subgroup.

The evaluated ultrasonographic characteristics included nodule size, solid structure, echogenicity, calcification status, margin characteristics, and TI-RADS category, when available. The presence of suspicious sonographic features, including solid structure, hypoechogenicity, microcalcification, irregular/suspicious margins, and TI-RADS 4, was recorded. Nodule size was defined as the maximum diameter measured on ultrasonography.

### 2.4. Histopathological Evaluation

Final histopathological examination of the surgical specimens was accepted as the reference standard. Histopathological diagnoses were obtained from postoperative pathology reports. Patients were classified into benign and malignant groups according to final pathology results. Cases diagnosed as non-invasive follicular thyroid neoplasm with papillary-like nuclear features (NIFTP) were classified within the benign group for statistical analyses in accordance with current recommendations.

### 2.5. Hematological Parameters and Inflammatory Indices

Complete blood count (CBC) parameters obtained during the preoperative evaluation were retrieved from the hospital electronic medical records. The NLR was calculated as the absolute neutrophil count divided by the absolute lymphocyte count, and the PLR was calculated as the platelet count divided by the absolute lymphocyte count. Hematological measurements were performed using an automated hematology analyzer as part of routine preoperative clinical assessment.

### 2.6. Statistical Analysis

Statistical analyses were performed using IBM SPSS Statistics software (version 27.0; IBM Corp., Armonk, NY, USA). Continuous variables were expressed as mean ± standard deviation (SD) or median (interquartile range [IQR]), according to data distribution, whereas categorical variables were presented as frequencies and percentages. Comparisons between benign and malignant groups were performed using the independent-samples *t*-test or the Mann–Whitney U test for continuous variables and the chi-square test or Fisher’s exact test for categorical variables, as appropriate. Variables with a *p*-value < 0.05 in the univariate analyses were entered into a multivariable logistic regression model to identify independent predictors of malignancy. Odds ratios (ORs) and corresponding 95% confidence intervals (CIs) were calculated. All statistical tests were two-sided, and a *p*-value < 0.05 was considered statistically significant.

## 3. Results

**Figure 1:**
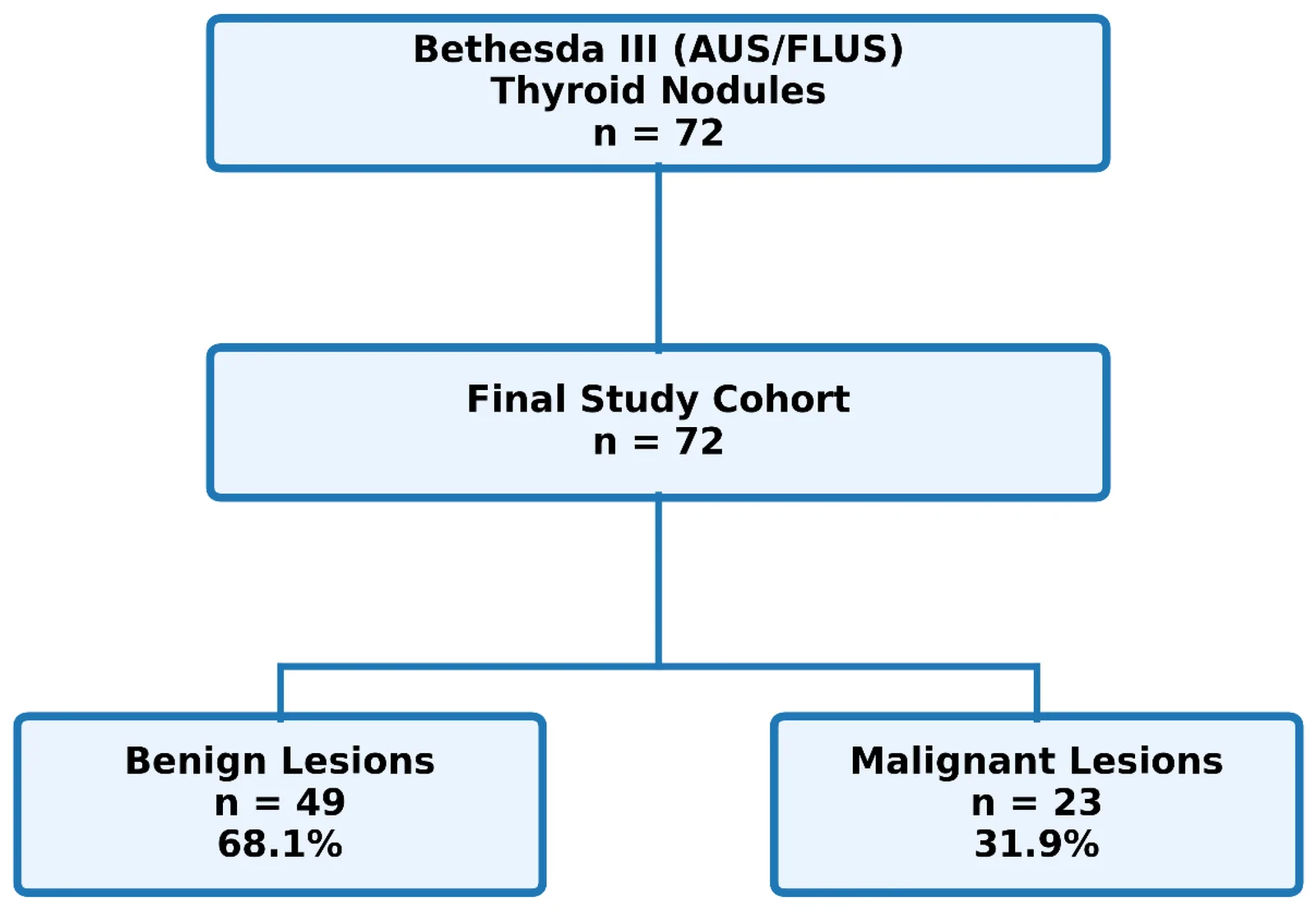
Flowchart of patient selection and final histopathological outcomes among patients with Bethesda category III (AUS/FLUS) thyroid nodules.

The study flowchart is shown in Figure 1. A total of 72 patients with Bethesda category III (AUS/FLUS) thyroid nodules who underwent thyroid surgery were included in the final analysis. Final histopathological examination revealed benign lesions in 49 patients (68.1%) and malignant lesions in 23 patients (31.9%).

The clinicopathological characteristics of the study population according to final histopathological diagnosis are summarized in Table 1. The mean age was comparable between the benign and malignant groups (46.5 ± 11.3 vs. 44.3 ± 12.5 years, *p* = 0.343). Similarly, no significant differences were observed regarding biopsied nodule size (26.9 ± 13.5 vs. 25.1 ± 17.5 mm, *p* = 0.304) or largest nodule diameter (27.0 ± 13.5 vs. 25.4 ± 18.5 mm, *p* = 0.284).

Female sex predominated in both groups without a significant association with malignancy (89.8% vs. 78.3%, *p* = 0.340). Likewise, multinodular goiter, Hashimoto thyroiditis, microcalcifications, and TI-RADS category did not differ significantly between benign and malignant nodules (all *p* > 0.05).

**Figure 2:**
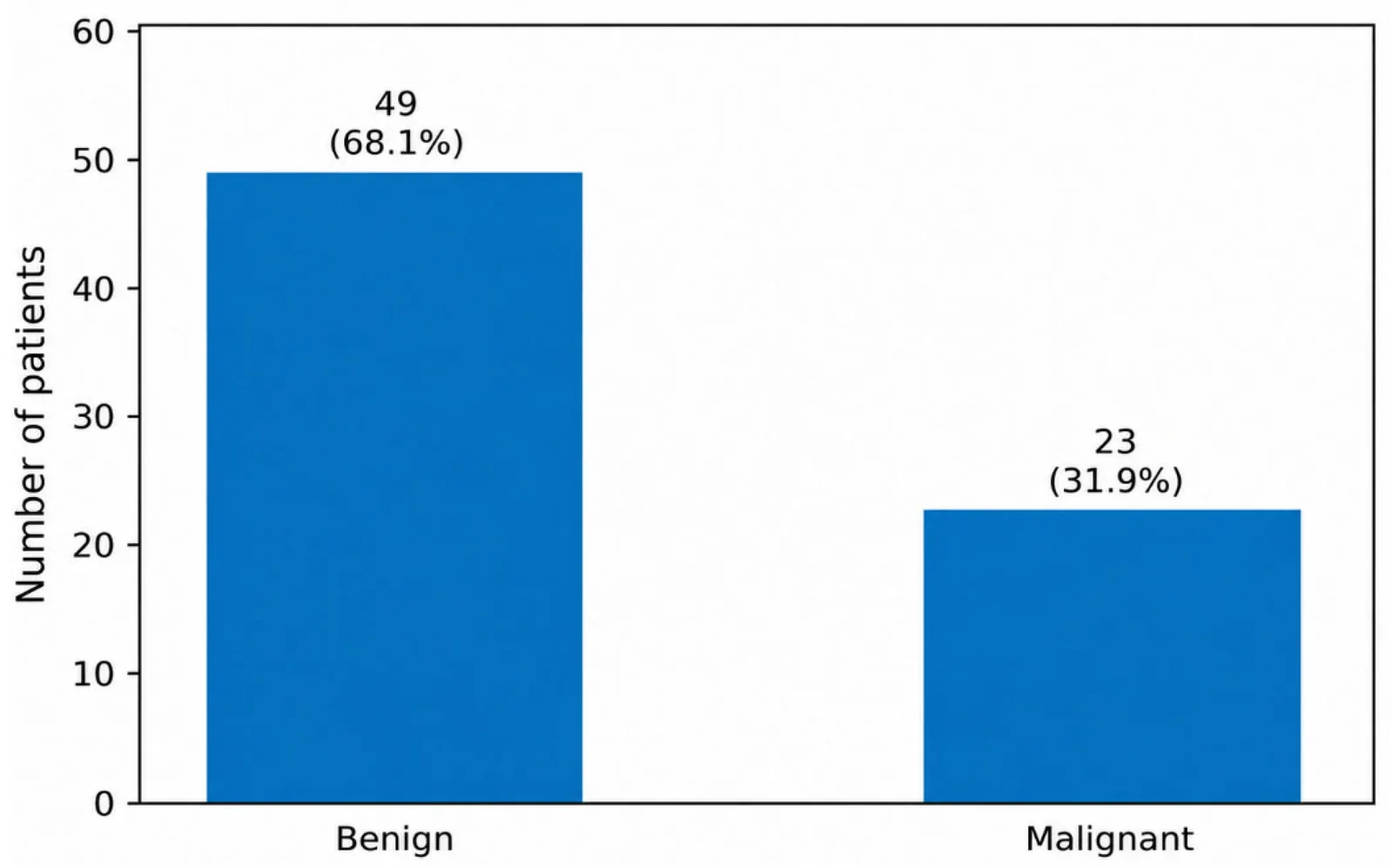
Distribution of final histopathological diagnoses. The figure shows the number and percentage of patients with benign (49/72, 68.1%) and malignant (23/72, 31.9%) histopathological outcomes.

The distribution of final histopathological outcomes is shown in Figure 2. Among the 72 surgically treated Bethesda III (AUS/FLUS) thyroid nodules, 49 lesions (68.1%) were classified as benign and 23 lesions (31.9%) as malignant. Papillary thyroid carcinoma accounted for the majority of malignant diagnoses, whereas nodular hyperplasia and follicular adenoma were the most common benign lesions. The detailed distribution of final histopathological diagnoses is presented in Table 2. Given the heterogeneous nature of Bethesda III cytology, the association between cytological atypia subtypes and malignant histopathological outcomes was subsequently evaluated.

Among 72 surgically treated Bethesda III thyroid nodules, final histopathological examination revealed malignancy in 23 patients (31.9%) and benign pathology in 49 patients (68.1%) (Table 3). No significant differences were observed between benign and malignant groups regarding age, nodule size, sex distribution, multinodular goiter, Hashimoto thyroiditis, or microcalcification status. None of the evaluated ultrasonographic characteristics demonstrated a statistically significant association with malignant histopathological outcomes.

**Figure 3:**
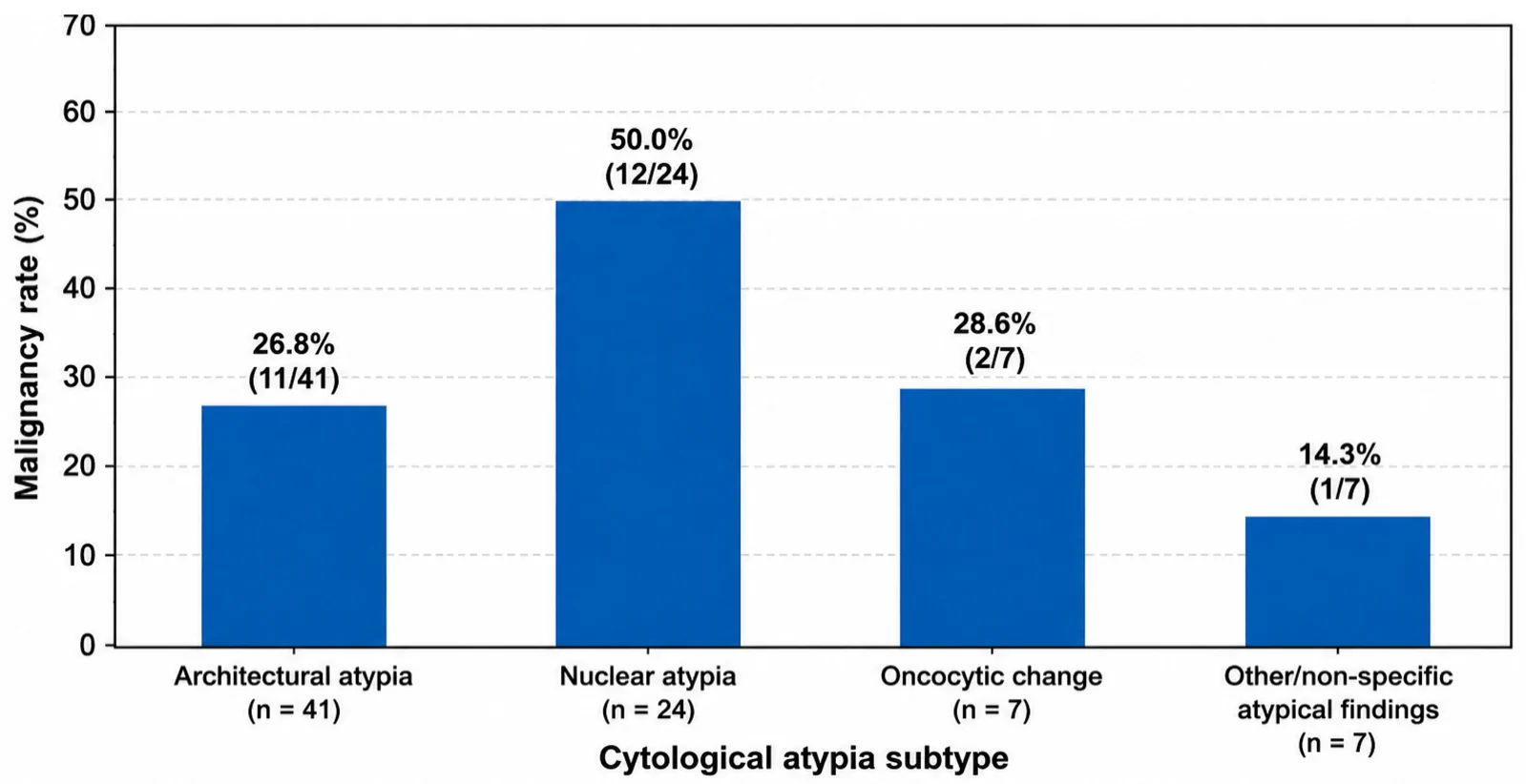
Malignancy rates according to cytological atypia subtype. Bars represent the percentage of malignant nodules within each cytological atypia subtype. Values above the bars indicate the malignancy rate (%) and the corresponding number of malignant cases/total cases (n/N). Nuclear atypia showed the highest malignancy rate (50.0%), followed by oncocytic change (28.6%), architectural atypia (26.8%), and other or non-specific atypical findings (14.3%).

The relationship between cytological atypia subtypes and malignancy risk is illustrated in Figure 3 and summarized in Table 4. Nodules with nuclear atypia demonstrated the highest malignancy rate (50.0%), compared with architectural atypia (23.5%), oncocytic change (28.6%), and other or non-specific atypical findings (14.3%). Although the overall distribution of atypia subtypes did not reach statistical significance (*p* = 0.125), the presence of nuclear atypia was significantly associated with malignant histopathological outcomes. Compared with all other cytological categories combined, nodules with nuclear atypia had a 3.36-fold higher risk of malignancy (OR = 3.364, *p* = 0.031).

The associations between inflammation-based hematological markers and final histopathological outcomes are summarized in Table 5. Patients with malignant nodules tended to have higher NLR and PLR values compared with those with benign lesions. However, neither NLR nor PLR differed significantly between the groups (NLR: 1.98 vs. 1.80, *p* = 0.319; PLR: 135.0 vs. 117.9, *p* = 0.398).

**Figure 4:**
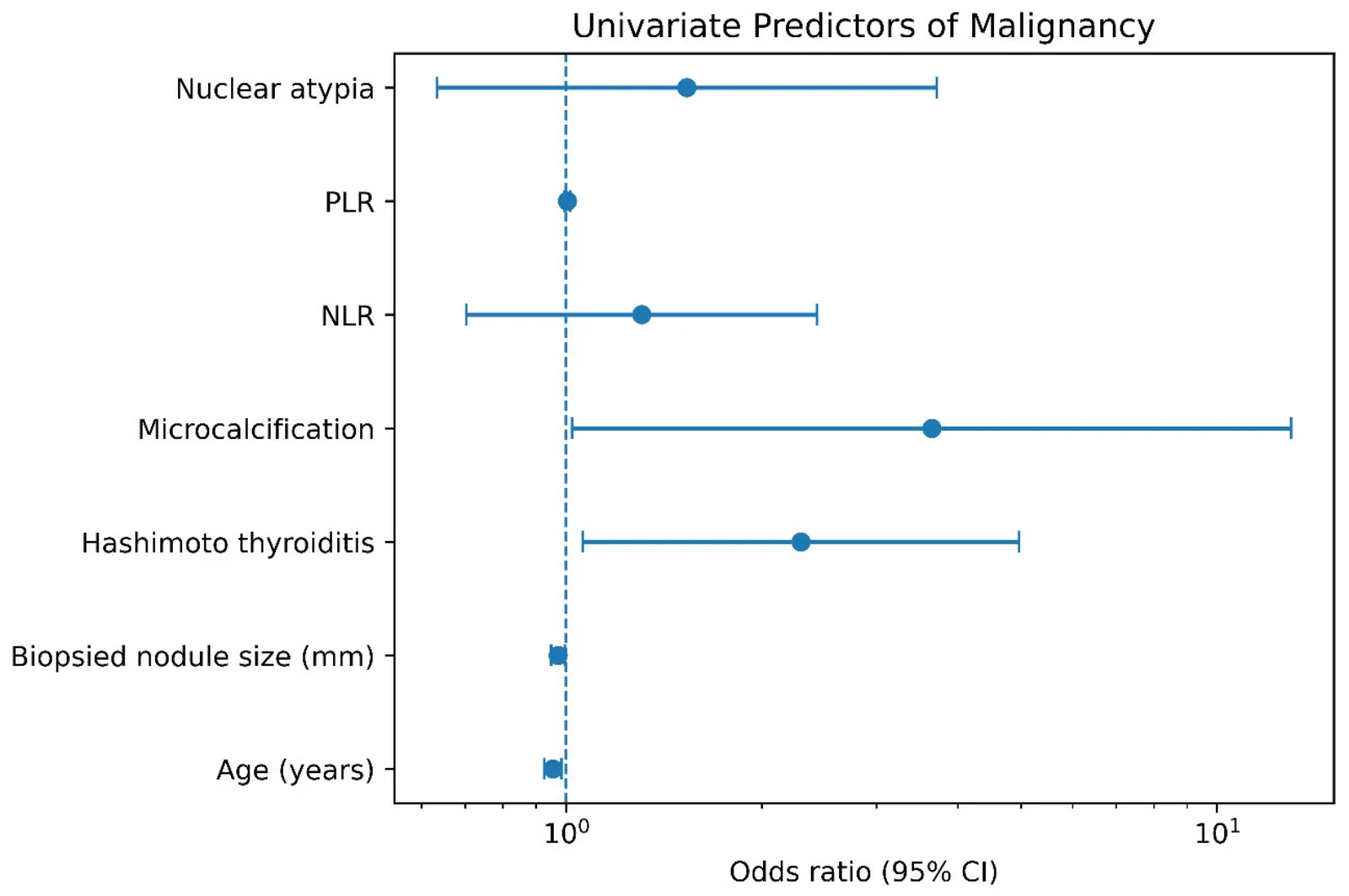
Univariate logistic regression analysis of predictors of malignancy in Bethesda III thyroid nodules. Dots represent odds ratios (ORs), and horizontal bars indicate 95% confidence intervals (CIs). The vertical dashed line denotes OR = 1.0. Nuclear atypia was the only significant predictor of malignancy.

The results of the univariate logistic regression analysis are presented in Table 6 and illustrated in Figure 4. Age, biopsied nodule size, largest nodule diameter, sex, multinodular goiter, Hashimoto thyroiditis, nodule composition, hypoechogenicity, microcalcifications, margin characteristics, and TI-RADS category were not significantly associated with malignant histopathological outcomes (all *p* > 0.05). Among the evaluated variables, only nuclear atypia was identified as a significant predictor of malignancy. Nodules exhibiting nuclear atypia had a 3.36-fold increased risk of malignancy compared with nodules lacking nuclear atypia (OR = 3.364, 95% CI: 1.182–9.570, *p* = 0.023). Although irregular or suspicious margins showed a trend toward an increased risk of malignancy (OR = 3.125, 95% CI: 0.730–13.370), the association did not reach statistical significance (*p* = 0.124). As shown in Figure 4, only the confidence interval for nuclear atypia did not cross the null value (OR = 1.0), supporting its role as the only significant univariate predictor of malignancy.

A multivariable logistic regression model adjusted for age and biopsied nodule size was performed to evaluate whether nuclear atypia remained associated with malignancy (Table 7). In this model, nuclear atypia remained a significant predictor of malignant histopathology (OR = 3.963, 95% CI: 1.298–12.094, *p* = 0.016). In contrast, age and biopsied nodule size were not independently associated with malignancy. Nuclear atypia was the only significant predictor of malignancy and remained significant after adjustment for age and nodule size.

## 4. Discussion

The present study evaluated the relationships between preoperative ultrasonographic features, inflammatory markers, cytological characteristics, and final histopathological outcomes in Bethesda category III (AUS/FLUS) thyroid nodules. The main finding was that nuclear atypia was the only independent predictor of malignancy, conferring an approximately fourfold increased risk after adjustment for age and biopsied nodule size. In contrast, none of the evaluated ultrasonographic features, including microcalcifications, hypoechogenicity, irregular/suspicious margins, or TI-RADS category, were significantly associated with malignant histopathological outcomes. Likewise, although NLR and PLR values were numerically higher in patients with malignant nodules, neither marker was significantly associated with malignancy. The absence of statistically significant associations should be interpreted with caution, as the small surgically treated cohort may have limited the statistical power to detect modest effects. Furthermore, the overall malignancy rate of 31.9% observed in our cohort is consistent with the range reported in the literature for surgically treated Bethesda III nodules and underscores the ongoing diagnostic challenges associated with this heterogeneous cytological category.

In the present study, age, sex, nodule size, multinodular goiter, Hashimoto thyroiditis, microcalcifications, and TI-RADS category were not significantly associated with malignancy. This finding is partly consistent with previous AUS/FLUS series showing that demographic variables such as age and sex may have limited predictive value in Bethesda III nodules [7,8]. Hong et al. [7] reported that age was not associated with malignancy and that the predictive value of sex and nodule size was inconsistent across studies. In another study, Hong et al. [8] found that age and sex were not significantly associated with malignancy risk in AUS/FLUS nodules, although malignant nodules tended to exhibit more unfavorable ultrasonographic features, particularly hypo- or anechogenicity. The 2015 American Thyroid Association (ATA) guidelines emphasize the importance of ultrasonographic risk stratification and identify features such as microcalcifications, irregular margins, marked hypoechogenicity, and taller-than-wide shape as suspicious findings associated with thyroid malignancy [9]. In contrast, other studies specifically focusing on Bethesda III nodules have reported significant associations between malignancy and suspicious ultrasonographic features such as microcalcifications, irregular margins, hypoechogenicity, and taller-than-wide shape [10,11,12]. The absence of significant ultrasonographic predictors in our cohort likely reflects the relatively small sample size, incomplete TI-RADS availability, interobserver variability inherent to retrospective ultrasound reporting, and selection bias resulting from the inclusion of only surgically treated Bethesda III nodules. Consequently, the predictive performance of ultrasonographic characteristics observed in this study may not accurately reflect their value in an unselected Bethesda III population. Furthermore, reliance on routine clinical ultrasound reports rather than centralized image review may have introduced measurement bias and reduced the ability to detect associations between sonographic features and malignancy. Therefore, the negative ultrasonographic findings should be interpreted cautiously, as they may partly reflect reporting variability and study design limitations rather than a true absence of biological association. Clinically, these findings suggest that ultrasonographic characteristics alone may be insufficient for risk stratification in surgically treated Bethesda III nodules and should be interpreted together with cytological findings.

Given the heterogeneous nature of Bethesda III cytology, we further investigated the impact of cytological atypia subtypes on malignancy risk. Nodules exhibiting nuclear atypia demonstrated the highest malignancy rate (50.0%), compared with architectural atypia (23.5%), oncocytic change (28.6%), and other atypical findings (14.3%). Although the overall distribution of atypia subtypes did not reach statistical significance, the presence of nuclear atypia was associated with a significantly increased risk of malignancy. In both univariate and multivariable logistic regression analyses, nuclear atypia emerged as the only significant predictor of malignant histopathological outcomes, conferring an approximately fourfold increased risk of malignancy. These findings are consistent with previous studies demonstrating that nuclear atypia is one of the strongest cytological predictors of malignancy within the Bethesda III category. Rosario et al. [13] reported significantly higher malignancy rates in AUS/FLUS nodules displaying nuclear atypia compared with those lacking nuclear atypical features, emphasizing the importance of nuclear changes in risk stratification. Similarly, VanderLaan et al. [14] demonstrated that cytological specimens exhibiting nuclear atypia were associated with a substantially increased likelihood of malignancy, supporting more careful clinical management in this subgroup. More recently, Şahin et al. [15] also identified cytological atypia as an important predictor of malignancy in AUS thyroid nodules. Our findings further reinforce the concept that the subclassification of Bethesda III nodules according to atypia type may provide clinically meaningful information beyond the conventional Bethesda categorization alone. Therefore, the negative findings for ultrasonographic characteristics, NLR, and PLR should be interpreted cautiously, as the limited sample size and the relatively small number of malignant cases may have reduced the ability to detect modest but clinically meaningful associations.

The potential role of systemic inflammatory markers in thyroid cancer has attracted increasing attention in recent years. Several studies have suggested that elevated NLR and PLR may reflect tumor-associated inflammation and could be associated with more aggressive clinicopathological characteristics in differentiated thyroid carcinoma. However, the evidence regarding their diagnostic utility in indeterminate thyroid nodules remains inconsistent. In agreement with our findings, some investigators have reported that NLR and PLR have limited value in distinguishing benign from malignant nodules in patients with indeterminate cytology and should not be used as standalone predictors of malignancy [16,17]. Conversely, other studies have demonstrated significantly higher NLR or PLR values in patients with thyroid cancer, suggesting a possible association between systemic inflammation and malignant transformation [18,19]. The lack of a significant association in our cohort may be explained by the relatively small sample size, the heterogeneous biological behavior of Bethesda III nodules, and the influence of various patient-related factors that can affect peripheral blood cell counts independently of thyroid pathology. Moreover, the relatively small number of malignant cases may have limited the statistical power to detect modest but clinically meaningful associations. Therefore, the negative findings for ultrasonographic characteristics, NLR, and PLR should be interpreted cautiously, as they may reflect a Type II error rather than a true absence of association. From a clinical perspective, these findings indicate that routinely available inflammatory markers such as NLR and PLR are unlikely to provide meaningful incremental value for preoperative risk stratification of Bethesda III nodules when used in isolation. Therefore, although NLR and PLR are inexpensive and readily available biomarkers, they should not be considered standalone tools for surgical decision-making; instead, they should be interpreted together with cytological findings, ultrasonographic characteristics, and the overall clinical context.

Interestingly, although irregular or suspicious margins showed a trend toward increased malignancy risk in our cohort, this association did not reach statistical significance. Previous studies have reported conflicting results regarding the predictive value of ultrasonographic findings in Bethesda III nodules, with some identifying suspicious ultrasound features as significant predictors of malignancy while others have found limited discriminatory value after adjustment for cytological characteristics [10,11,12]. Taken together, our findings suggest that cytological subclassification, particularly the identification of nuclear atypia, provides greater value for preoperative risk stratification than conventional ultrasonographic characteristics or systemic inflammatory markers in patients with Bethesda III thyroid nodules.

### Strengths and Limitations

This study has several strengths. First, all patients had Bethesda category III (AUS/FLUS) cytology and underwent surgical treatment, allowing direct comparison between preoperative findings and definitive histopathological diagnoses. Second, clinical, ultrasonographic, cytological, and inflammation-based hematological parameters were evaluated simultaneously, providing a comprehensive assessment of factors potentially associated with malignancy. Third, the study examined cytological atypia subtypes and demonstrated that nuclear atypia remained an independent predictor of malignancy after adjustment for age and nodule size, supporting the clinical value of Bethesda III subclassification.

Several limitations should also be acknowledged. The retrospective single-center design may limit the generalizability of the findings and introduce the possibility of selection bias. The relatively small sample size reduced statistical power and may have limited the detection of significant associations for some ultrasonographic and hematological variables. Consequently, the possibility of a Type II error cannot be excluded, and the absence of statistically significant associations should not be interpreted as definitive evidence of the absence of clinically relevant relationships. In addition, only surgically treated Bethesda III nodules were included, which may have resulted in a higher prevalence of malignancy than would be expected in an unselected Bethesda III population. This selection bias may also have influenced the observed associations between preoperative variables and malignancy, thereby limiting the generalizability of our findings. Ultrasonographic findings were obtained from routine clinical reports rather than centralized image review, and interobserver variability could not be assessed. Accordingly, measurement bias related to retrospective ultrasound interpretation cannot be excluded. Furthermore, because standardized archived ultrasound images were not available for all patients, a blinded re-evaluation by a single experienced radiologist was not feasible. Therefore, the lack of significant associations between ultrasonographic features and malignancy may, at least in part, reflect interobserver variability and measurement bias rather than a true absence of biological association. Because of the limited sample size and the relatively small number of malignant cases, additional subgroup analyses according to nodule size or TI-RADS category were not performed, as these analyses would have been statistically underpowered and unlikely to provide reliable conclusions. Finally, molecular testing was not available, precluding comparison of cytological, ultrasonographic, and molecular predictors of malignancy.

In conclusion, the present study demonstrated that nuclear atypia is an independent predictor of malignancy in Bethesda category III (AUS/FLUS) thyroid nodules. In contrast, conventional ultrasonographic characteristics, NLR, and PLR showed limited value in distinguishing benign from malignant lesions in this surgically treated cohort. These findings support the clinical importance of cytological subclassification within the Bethesda III category and suggest that the identification of nuclear atypia may improve preoperative risk stratification and surgical decision-making. Nevertheless, because the study included only surgically treated patients, selection bias is unavoidable, and the observed malignancy rate may not reflect that of the overall Bethesda III population. Therefore, these findings should be interpreted with caution and regarded as hypothesis-generating rather than definitive. Further large prospective multicenter studies incorporating standardized ultrasonographic assessment and molecular markers are warranted to validate these findings and refine risk prediction models for indeterminate thyroid nodules.

## Figures and Tables

**Table 1:** Clinicopathological characteristics of patients according to final histopathological diagnosis.

Variable	Benign (*n* = 49)	Malignant (*n* = 23)	*p* Value
Age (years)	46.5 ± 11.3	44.3 ± 12.5	0.343
Biopsied nodule size (mm)	26.9 ± 13.5	25.1 ± 17.5	0.304
Largest nodule diameter (mm)	27.0 ± 13.5	25.4 ± 18.5	0.284
Female sex, *n* (%)	44 (89.8)	18 (78.3)	0.340
Multinodular goiter, *n* (%)	22 (44.9)	11 (47.8)	1.000
Hashimoto thyroiditis, *n* (%)	7 (14.3)	4 (17.4)	0.734
Microcalcification, *n* (%)	3 (6.1)	3 (13.0)	0.382
TI-RADS 3, *n* (%)	26 (89.7) *	12 (85.7) *	1.000
TI-RADS 4, *n* (%)	3 (10.3) *	2 (14.3) *	

* Percentages were calculated based only on patients with available TI-RADS classification. TI-RADS classification was available for 43 of the 72 patients (59.7%), including 29 of 49 patients in the benign group and 14 of 23 patients in the malignant group; the remaining 29 patients (40.3%) had no documented TI-RADS classification in the original ultrasound reports.

**Table 2:** Final histopathological diagnoses.

Histopathological Diagnosis	*n* (%)
Papillary thyroid carcinoma *	21 (29.2)
Borderline follicular-patterned neoplasm **	1 (1.4)
Other malignant lesion	1 (1.4)
Nodular hyperplasia	25 (34.7)
Follicular adenoma	16 (22.2)
Hürthle cell adenoma/neoplasm	4 (5.6)
Other benign lesions	4 (5.6)
Total	72 (100)

* Including papillary thyroid carcinoma variants (classical, follicular variants, oncocytic variants, microcarcinoma, etc.). ** Well-differentiated tumor of uncertain malignant potential (WDT-UMP)/similar borderline lesions.

**Table 3:** Ultrasonographic features according to final pathology.

Variable	Benign (*n* = 49)	Malignant (*n* = 23)	*p* Value
Age (years)	46.5 ± 11.3	44.3 ± 12.5	0.343
Biopsied nodule size (mm)	26.9 ± 13.5	25.1 ± 17.5	0.304
Largest nodule diameter (mm)	27.0 ± 13.5	25.4 ± 18.5	0.284
Female sex, *n* (%)	44 (89.8)	18 (78.3)	0.340
Multinodular goiter, *n* (%)	22 (44.9)	11 (47.8)	1.000
Hashimoto thyroiditis, *n* (%)	7 (14.3)	4 (17.4)	0.734
Microcalcification, *n* (%)	3 (6.1)	3 (13.0)	0.382

**Table 4:** Cytological atypia and risk of malignancy.

Cytological Atypia	Benign (*n* = 49)	Malignant (*n* = 23)	Malignancy Rate (%)	*p* Value
Architectural atypia	26 (53.1)	8 (34.8)	23.5	0.125
Nuclear atypia	12 (24.5)	12 (52.2)	50	
Oncocytic change	5 (10.2)	2 (8.7)	28.6	
Other/none	6 (12.2)	1 (4.3)	14.3	

Nuclear atypia vs. all other categories: OR = 3.364, Fisher’s exact *p* = 0.031.

**Table 5:** Comparison of inflammatory markers according to final histopathological diagnosis.

Variable	Benign (*n* = 49)	Malignant (*n* = 23)	Mann–Whitney U	*p* Value
NLR	1.80 (1.30–2.20)	1.98 (1.52–2.40)	646.5	0.319
PLR	117.9 (105.0–148.2)	135.0 (104.0–175.0)	634.0	0.398

Data are presented as median (interquartile range). Comparisons between groups were performed using the Mann–Whitney U test. NLR, neutrophil-to-lymphocyte ratio; PLR, platelet-to-lymphocyte ratio.

**Table 6:** Univariate logistic regression analysis for predictors of malignancy.

Variable	OR	95% CI	*p* Value
Age (years)	0.983	0.941–1.027	0.439
Biopsied nodule size (mm)	0.992	0.958–1.027	0.642
Largest nodule diameter (mm)	0.992	0.957–1.029	0.673
Female sex	0.409	0.105–1.587	0.196
Multinodular goiter	1.125	0.373–3.391	0.834
Hashimoto thyroiditis	1.257	0.319–4.956	0.744
Solid structure	0.591	0.205–1.704	0.330
Hypoechogenicity	1.179	0.390–3.566	0.771
Microcalcification	2.25	0.409–12.381	0.351
Irregular/suspicious margin	3.125	0.730–13.370	0.124
TI-RADS 4	1.444	0.213–9.808	0.707
NLR	1.310	0.700–2.430	0.397
PLR	1.010	0.990–1.020	0.246
Nuclear atypia	3.364	1.182–9.570	0.023

OR, odds ratio; CI, confidence interval; NLR, neutrophil-to-lymphocyte ratio; PLR, platelet-to-lymphocyte ratio. For continuous variables (age and nodule size), ORs represent the odds of malignancy associated with a one-unit increase (1 year for age and 1 mm for nodule size).

**Table 7:** Multivariable logistic regression analysis adjusted for age and biopsied nodule size.

Variable	OR	95% CI	*p* Value
Nuclear atypia	3.963	1.298–12.094	0.016
Age (years)	0.969	0.924–1.016	0.188
Biopsied nodule size (mm)	0.995	0.960–1.031	0.779

OR, odds ratio; CI, confidence interval. Age was analyzed per 1-year increase and nodule size per 1-mm increase.

## Data Availability

The datasets used and/or analyzed during the current study are available from the corresponding author on reasonable request.

## References

[1] Guth S., Theune U., Aberle J., Galach A., Bamberger C.M. (2009). Very high prevalence of thyroid nodules detected by high frequency (13 MHz) ultrasound examination. Eur. J. Clin. Investig..

[2] Li G., Li R., Zhong J., Chen W., Shuai J., Chen M., Deng F., Wei T., Tang H., Li Z. (2025). A multicenter cohort study of thyroidectomy-related decision regret in patients with low-risk papillary thyroid microcarcinoma. Nat. Commun..

[3] Cibas E.S., Ali S.Z. (2017). The 2017 Bethesda System for Reporting Thyroid Cytopathology. Thyroid.

[4] Ali S.Z., Baloch Z.W., Cochand-Priollet B., Schmitt F.C., Vielh P., VanderLaan P.A. (2023). The 2023 Bethesda System for Reporting Thyroid Cytopathology. Thyroid.

[5] Ali S.Z., Cibas E.S. (2023). The Bethesda System for Reporting Thyroid Cytopathology: Definitions, Criteria, and Explanatory Notes.

[6] Templeton A.J., McNamara M.G., Šeruga B., Vera-Badillo F.E., Aneja P., Ocaña A., Leibowitz-Amit R., Sonpavde G., Knox J.J., Tran B. (2014). Prognostic role of neutrophil-to-lymphocyte ratio in solid tumors: A systematic review and meta-analysis. J. Natl. Cancer Inst..

[7] Hong S.H., Lee H., Cho M.S., Lee J.E., Sung Y.A., Hong Y.S. (2018). Malignancy Risk and Related Factors of Atypia of Undetermined Significance/Follicular Lesion of Undetermined Significance in Thyroid Fine Needle Aspiration. Int. J. Endocrinol..

[8] Hong I.K., Kim J.H., Cho Y.U., Park S.Y., Kim S.J. (2016). Clinicopathological factors increased the risk of malignancy in thyroid nodules with atypical or follicular lesions of undetermined significance (AUS/FLUS) risk factor of malignancy in thyroid nodule with AUS/FLUS. Ann. Surg. Treat. Res..

[9] Haugen B.R., Alexander E.K., Bible K.C., Doherty G.M., Mandel S.J., Nikiforov Y.E., Pacini F., Randolph G.W., Sawka A.M., Schlumberger M. (2016). 2015 American Thyroid Association Management Guidelines for Adult Patients with Thyroid Nodules and Differentiated Thyroid Cancer: The American Thyroid Association Guidelines Task Force on Thyroid Nodules and Differentiated Thyroid Cancer. Thyroid.

[10] Alshahrani A.S., Alamri A.S., Balkhoyor A.H., Mahzari M.M., Alshieban S.S., Majed P.M. (2021). The Prediction of Malignancy Risk in Thyroid Nodules Classified as Bethesda System Category III (AUS/FLUS) and the Role of Ultrasound Finding for Prediction of Malignancy Risk. Cureus.

[11] Liu X., Wang J., Du W., Dai L., Fang Q. (2022). Predictors of Malignancy in Thyroid Nodules Classified as Bethesda Category III. Front. Endocrinol..

[12] Alyusuf E.Y., Alhmayin L., Albasri E., Enani J., Altuwaijri H., Alsomali N., Arafah M.A., Alyusuf Z., Jammah A.A., Ekhzaimy A.A. (2024). Ultrasonographic predictors of thyroid cancer in Bethesda III and IV thyroid nodules. Front. Endocrinol..

[13] Rosario P.W. (2014). Thyroid nodules with atypia or follicular lesions of undetermined significance (Bethesda Category III): Importance of ultrasonography and cytological subcategory. Thyroid.

[14] VanderLaan P.A., Marqusee E., Krane J.F. (2011). Clinical outcome for atypia of undetermined significance in thyroid fine-needle aspirations: Should repeated FNA be the preferred initial approach?. Am. J. Clin. Pathol..

[15] Şahin S., Pehlevan Özel H., Yüksek Y.N. (2025). Predictive Factors for Malignancy in Atypiai of Undetermined Significance (AUS) Thyroid Nodules: A Comprehensive Retrospective Analysis. Curr. Oncol..

[16] Kim S.M., Kim E.H., Kim B.H., Kim J.H., Bin Park S., Nam Y.J., Ahn K.H., Oh M.Y., Kim W.J., Jeon Y.K. (2015). Association of the Preoperative Neutrophil-to-ymphocyte Count Ratio and Platelet-to-Lymphocyte Count Ratio with Clinicopathological Characteristics in Patients with Papillary Thyroid Cancer. Endocrinol. Metab..

[17] Manatakis D.K., Tseleni-Balafouta S., Balalis D., Soulou V.N., Korkolis D.P., Sakorafas G.H., Plataniotis G., Gontikakis E. (2017). Association of Baseline Neutrophil-to-Lymphocyte Ratio with Clinicopathological Characteristics of Papillary Thyroid Carcinoma. Int. J. Endocrinol..

[18] Liu C.L., Lee J.J., Liu T.P., Chang Y.C., Hsu Y.C., Cheng S.P. (2013). Blood neutrophil-to-lymphocyte ratio correlates with tumor size in patients with differentiated thyroid cancer. J. Surg. Oncol..

[19] Gong W., Yang S., Yang X., Guo F. (2016). Blood preoperative neutrophil-to-lymphocyte ratio is correlated with TNM stage in patients with papillary thyroid cancer. Clinics.

